# Oral Supplementation of Glucosamine Fails to Alleviate Acute Kidney Injury in Renal Ischemia-Reperfusion Damage

**DOI:** 10.1371/journal.pone.0161315

**Published:** 2016-08-24

**Authors:** Marc Johnsen, Martin Richard Späth, Martin S. Denzel, Heike Göbel, Torsten Kubacki, Karla Johanna Ruth Hoyer, Yvonne Hinze, Thomas Benzing, Bernhard Schermer, Adam Antebi, Volker Burst, Roman-Ulrich Müller

**Affiliations:** 1 Department II of Internal Medicine and Center for Molecular Medicine, University of Cologne, Kerpener Str. 62, 50937, Cologne, Germany; 2 Max Planck Institute for Biology of Ageing, Joseph-Stelzmann-Str. 9b, 50931, Cologne, Germany; 3 Cologne Excellence Cluster on Cellular Stress Responses in Aging-Associated Diseases, University of Cologne, Cologne, Germany; 4 Systems Biology of Ageing Cologne, University of Cologne, Cologne, Germany; 5 Institute for Pathology, Diagnostic and Experimental Nephropathology Unit, University of Cologne, Cologne, Germany; University of Edinburgh MRC Centre for Inflammation Research, UNITED KINGDOM

## Abstract

Acute kidney injury is a leading contributor to morbidity and mortality in the ageing population. Proteotoxic stress response pathways have been suggested to contribute to the development of acute renal injury. Recent evidence suggests that increased synthesis of N-glycan precursors in the hexosamine pathway as well as feeding of animals with aminosugars produced in the hexosamine pathway may increase stress resistance through reducing proteotoxic stress and alleviate pathology in model organisms. As feeding of the hexosamine pathway metabolite glucosamine to aged mice increased their life expectancy we tested whether supplementation of this aminosugar may also protect mice from acute kidney injury after renal ischemia and reperfusion. Animals were fed for 4 weeks ad libitum with standard chow or standard chow supplemented with 0.5% N-acetylglucosamine. Preconditioning with caloric restriction for four weeks prior to surgery served as a positive control for protective dietary effects. Whereas caloric restriction demonstrated the known protective effect both on renal function as well as survival in the treated animals, glucosamine supplementation failed to promote any protection from ischemia-reperfusion injury. These data show that although hexosamine pathway metabolites have a proven role in enhancing protein quality control and survival in model organisms oral glucosamine supplementation at moderate doses that would be amenable to humans does not promote protection from ischemia-reperfusion injury of the kidney.

## Introduction

Acute kidney injury (AKI) is a frequent complication in hospitalized patients and common diseases like diabetes mellitus or hypertension are risk factors. AKI leads to increased morbidity and mortality and represents a growing economical burden [1]. Frequently, it can progress to chronic kidney disease (CKD) ultimately necessitating renal replacement therapy. To date, there are no therapeutic approaches that are able to modify the natural course of AKI. Regarding the urgent need for such strategies induction of mechanisms that mediate cellular stress resistance such as longevity pathways have become a central research focus [2,3]. A number of mechanisms prolonging life have been shown to convey increased stress resistance in a multitude of organs and species. As an example, there is ample evidence that dietary restriction and hypoxia signaling both lead to longevity and protection from organ failure [2–5]. Unfortunately, none of these approaches have made their way to the clinical setting yet. This is partly due to the fact that many animal models that were used depend on either genetic modifications or modes of preconditioning such as the exposure to hypoxia or caloric restriction that are difficult to translate to the clinic.

Posttranslational modifications of proteins by O-linked coupling of N-acetylglucosamine (GlcNAc) have been implicated to play a role in cell survival and elevated stress resistance [6]. The precursor of O- and N-glycans, UDP-N-acetylglucosamine (UDP-GlcNAc), is provided by the hexosamine pathway (HP). Recent evidence revealed that, both, activation of the HP or feeding of the resulting aminosugars leads to robust lifespan extension in mice and nematodes [7,8]. It was shown that acute induction of the hexosamine pathway through the spliced transcription factor Xbp1s, which acts upstream of the HP, leads to organ protection in a mouse model of cardiac ischemia-reperfusion injury (IR) [9]. Moreover, Jensen et al. suggested that the protective effects of remote ischemic (RIPC) and ischemic preconditioning (IPC) are due to increased circulating GlcNAc levels and O-GlcNAcylation, as well [10]. The positive effects of the HP do not appear to be limited to the heart since GlcNAc administration before and especially after ischemia of the eye has been shown to mediate retinal cell survival [11].

In this study we investigated whether GlcNAc supplementation can serve as a novel treatment strategy to prevent AKI in a murine ischemia-reperfusion model. Such an approach would be of high interest, since GlcNAc is already available as a dietary supplement and there is little concern about potential side effects.

## Material and Methods

### Ethical statement

All animal procedures were approved by the “Landesamt für Natur, Umwelt und Verbraucherschutz NRW”(LANUV 84–02.04.2013.A158). Mice were obtained from Charles River (Sulzfeld, Germany). Standard and GlcNAc enriched chow were obtained from Ssniff Spezialdiäten GmbH (Soest, Germany). Animals were monitored on a daily basis and checked for abnormal behavior and signs of stress. Furthermore, a score sheet for premature endpoints was used. The sheet contained scores for weight loss, reduced activity and poor appearance. Mice on GlcNAc enriched chow and the corresponding controls were weighed weekly. Animals received 0.6 ml of normal saline containing 0.2 mg tramadol subcutaneously after surgery to compensate for fluid loss and to reduce pain. Mice were sacrificed by exsanguination, performed under ketamine-xylazine anesthesia administered intraperitoneally (i.p.).

### Animals

Male, wild type C57BL/6J mice were kept under identical specific pathogen free (SPF) conditions in group-cages with 5 animals. After surgery mice were transferred to single cages to prevent mutilation of the surgical wound by fellow mice. Animals were fed for 4 weeks (the age was 13 to 14 weeks at time of surgery) ad libitum with standard chow or standard chow supplemented with 0.5% GlcNAc. Given an average food consumption of 4.5 g this roughly equals 22.5 mg GlcNAc per day. Another group of mice (11 to 12 weeks of age) received 250 μl of a 10% GlcNAc solution (25 mg) or the same amount of PBS 24 hours and 2 hours prior to surgery via gavage. Ultimately, a third and fourth group of mice (11 to 12 weeks of age) were injected intraperitoneally (i.p.) with 20 mg of GlcNAc either 60 min before surgery or directly at the onset of reperfusion.

For preconditioning with caloric restriction mice received 60% of normal calorie intake for four weeks prior to surgery.

### Renal ischemia-reperfusion model

We used a warm renal ischemia-reperfusion model as described by Khan et al. [12] with slight modifications. Surgery was performed under ketamine-xylazine anesthesia administered i.p. Briefly, mice were placed on a temperature-controlled heating pad. Right nephrectomy was performed after midline abdominal incision. Afterwards the left renal pedicle was mobilized and clamped for 40 minutes with an atraumatic micro-vascular clamp. After visual change in kidney color to dark purple the abdomen was covered with a compress soaked in normal saline. Restoration of blood flow was inspected visually and the abdominal wound was closed in two layers. Animals were sacrificed 24 hours after reperfusion.

Sham animals underwent a right nephrectomy and mobilization of the left renal pedicle without clamping. 40 minutes later the abdominal wound was closed in two layers and after 24 h animals were sacrificed.

### Functional measurements

Blood was harvested in heparin coated 1 ml syringes by exsanguination from the right ventricle. After resting for 30 minutes at room temperature, blood was centrifuged at 2500 g for 10 minutes at room temperature. Plasma urea and creatinine values were analyzed in the central laboratory of the University Hospital of Cologne utilizing a Cobas C 702 (Roche Diagnostics). Creatinine was quantified with an enzymatic test ultimately measuring a quinoneimine colorant. Urea was quantified with an enzymatic test detecting the decrease in extinction due to consumption of NADH.

Animals used for baseline measurement of serum creatinine, urea and GlcNAc were sacrificed at the same time the surgery would have been planned.

### Plasma GlcNAc levels

LC/MS/MS Analysis for the determination of plasma GlcNAc levels were conducted as described in Denzel et al. [8]. Briefly, absolute quantification of GlcNAc was done using an Acquity UPLC and XevoTM TQ (Waters). The compound was separated on a Merck zic-HILIC column 3.5μm, 200A, PEEK (50 x 2.1 mm) at 40°C. The two mobile phases consist of (A) 10mM ammonium acetate (pH 5.0) and (B) acetonitrile. With GlcNAc eluting at 0.6 min a standard calibration curve was calculated using following concentrations: 25, 50, 100, 150, 200, 250, 300, 500, 750 ng/mL (daily fresh diluted with 50% M9/MeOH individually from stock solutions 100 μg/ml). Correlation coefficient: r < 0.990; response type: external standard, area; curve type linear; weighting 1/x. The following MRM transitions were used for GlcNAc m/z 219.9 (M-H+)- to 119.01 (quantifier) collision energy 8V, m/z 219.9 to 58.99 (qualifier) collision 14V, m/z 219.9 to 100.81 (qualifier) collision 10V, cone was in all cases 16V. The peak integrations were corrected manually, if necessary. A new calibration curve as quality control standards were used during sample analysis and showed between 0.5% and 40% deviation respectively. Blanks after the standards and each sample with 4 technical replicates, quality control and sample batch proved to be sufficient. No carry over was detected.

### Histopathology

Formalin-fixed paraffin sections (2 μm) were stained with Periodic Acid-Schiff (PAS). 5 visual fields per section were analyzed at a 200x magnification. Pictures were taken with a Leica Slidescanner SCN4000. Sections were analyzed for acute tubular damage by an experienced pathologist with expertise in renal pathology in a blinded manner and categorized based on the presence or absence of vacuolization, epithelial flattening, loss of brush border, loss of nuclei and necrosis with a score similar to the ones used by Tirapelli et al. and Goujon et al. [13,14]. Results were graded 0 to 4 according to the affected area. (1: 0–25%, 2: 25–50%, 3: 50–75%, 4: 75–100%).

### Terminal deoxynucleotidyl transferase dUTP nick end labeling (TUNEL) staining

Staining was performed on formalin-fixed paraffin sections (2 μm) using the DeadEnd Fluorometric TUNEL System (Promega, USA) according to the manufacturer’s protocol. For documentation, pictures were taken with a Zeiss Meta 710 confocal microscope.

### Statistical analysis

Statistical analysis was done with GraphPad Prism Software V6.0c. Results are presented as means ± SEM. For each experiment, at least three biological replicates were examined. To calculate differences between multiple groups we used one-way ANOVA and a Tukey’s multiple comparisons test, if a Gauss distribution was assumed, and a Kruskall-Wallis test and a Dunn’s multiple comparisons test, if not. For comparing two groups the Mann-Whitney test and Kolmogorov-Smirnov test were utilized. Significance of weight differences between GlcNAc and control animals was calculated with multiple t-tests.

## Results

### Baseline urea and creatinine values in animals treated with GlcNAc or GlcN and control mice

**Fig 1:**
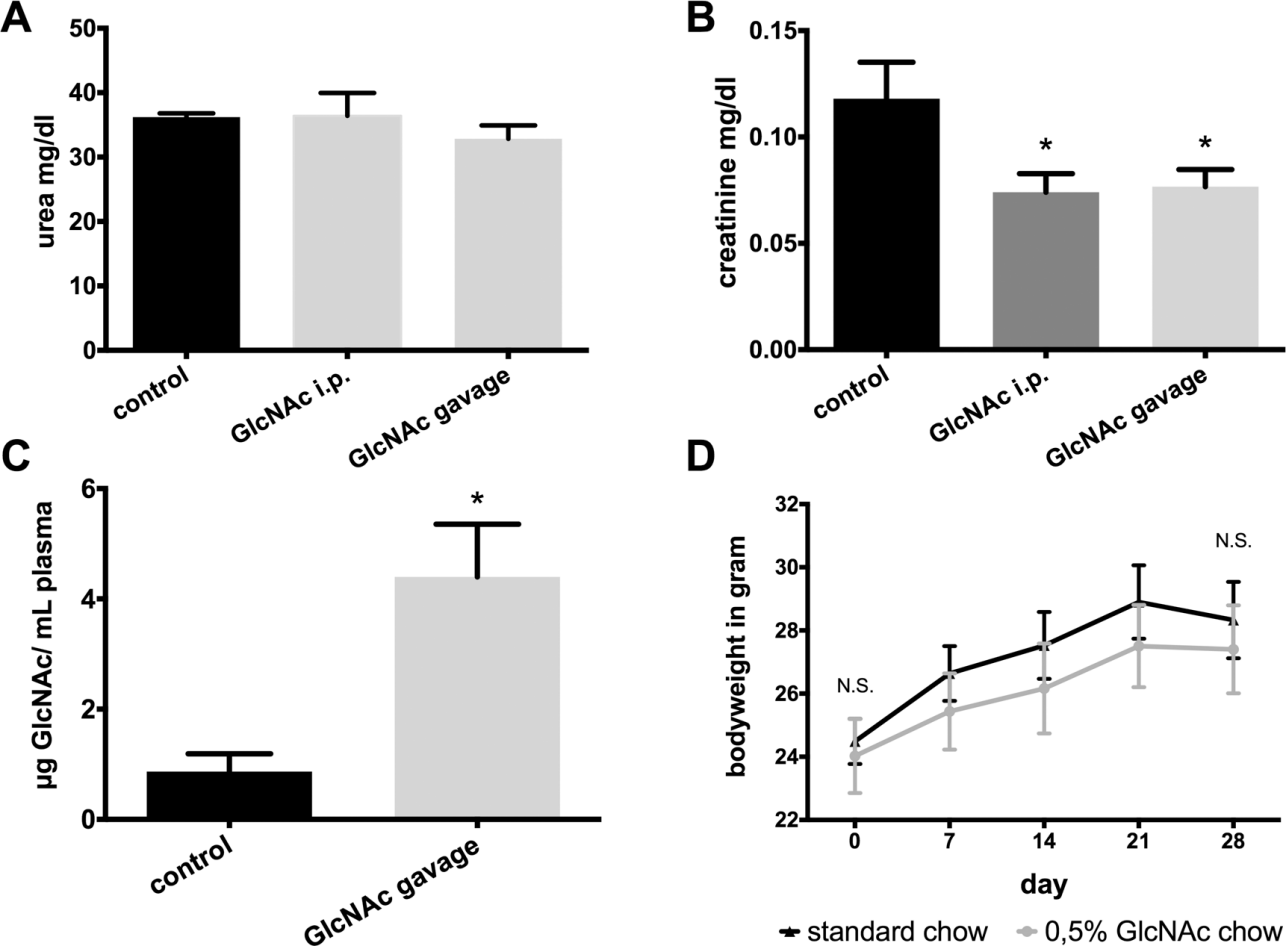
Baseline characteristics of 14 weeks old male BL6 mice treated with GlcNAc or kept on ad libitum standard chow. A) and B) Baseline creatinine and urea values (control n = 5, GlcNAc i.p. n = 5, GlcNAc gavage n = 6) C) Plasma GlcNAc levels 2 hours after gavage of 250 μl of a 10% GlcNAc solution (n = 6) and in control animals with ad libitum access to water and standard chow (n = 5). D) Weight curves of control mice (n = 10) and mice kept on a 4 weeks ad libitum diet of chow enriched with 0.5% GlcNAc (n = 9).

To examine, if GlcNAc supplementation per se affects kidney function, 14 weeks old male BL6 mice were treated with GlcNAc by either intraperitoneal injection or gavage and serum urea and creatinine were assessed. As compared to control animals without supplementation there was no statistically significant difference for serum urea between the different groups (Fig 1A). Interestingly, GlcNAc administration induced a slight but significant reduction of baseline creatinine (Fig 1B).

### Serum GlcNAc levels after application of GlcNac or GlcN

Next, we wanted to confirm that GlcNAc application led to an increase in plasma GlcNAc levels. Therefore, GlcNAc levels were measured with LCMS. As expected, short term oral administration of GlcNAc led to a significant elevation of GlcNAc levels in plasma (Fig 1C).

### Effect of GlcNAc on weight gain in ad libitum fed animals

GlcNAc has a similar taste to sucrose with only half of its sweetness [15]. For mice fed with a GlcNAc supplemented diet a potentially increased food uptake and, hence, weight gain may significantly impact on organ stress resistance given our knowledge about dietary restriction mediated organ protection [16]. After four weeks of treatment there was no significant difference in weight gain between controls and GlcNAc fed animals (Fig 1D).

### Effect of GlcNAc supplementation on histopathological signs of tubular damage

**Fig 2:**
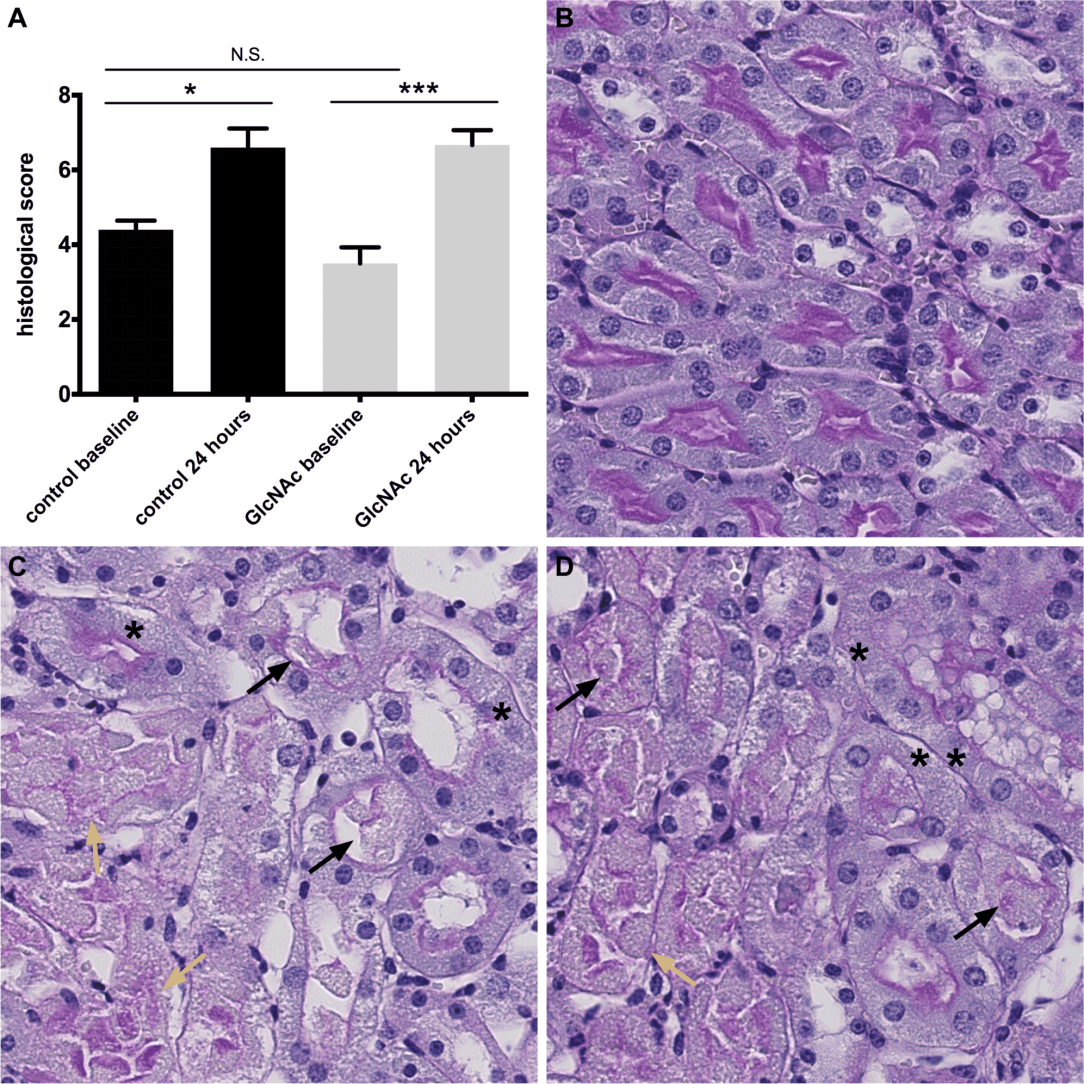
Histology before and after IR. A) Damage score after inspection of 5 HPF at the corticomedullary border (control baseline n = 5, control 24 hours n = 6, GlcNAc baseline n = 11, GlcNAc 24 hours n = 14). Sections were evaluated in a blinded manner by an experienced nephropathologist. B)–D) Representative PAS stainings from kidneys before and after IR. (X 200) B) Uninephrectomy section from undamaged kidney. C) and D) Kidney sections 24 hours after the end of ischemia. Asterix marks vanishing or missing nuclei. Black arrows mark tubular casts consisting of tubular cells. Yellow arrows show necrotic areas and denuded tubuli with regions just consisting of naked basement membrane. C) Kidney from animal treated with twice oral gavage of 10% GlcNAc solution before IR. (D) Kidney from control animal with twice oral gavage of PBS before IR.

We used the histopathological score, similar to the one described in Tirapelli et al. and Goujon et al. [13,14], to assess the extend of renal damage after ischemia-reperfusion. Tubular damage at the corticomedullary border was similar in GlcNAc-treated and control animals. Furthermore, 24 hours after reperfusion we observed brush border loss, tubular flattening and casts along with necrotic areas, mainly in proximal tubuli, in all groups (Fig 2).

### Effect of GlcNAc supplementation on apoptosis after ischemia-reperfusion injury

**Fig 3:**
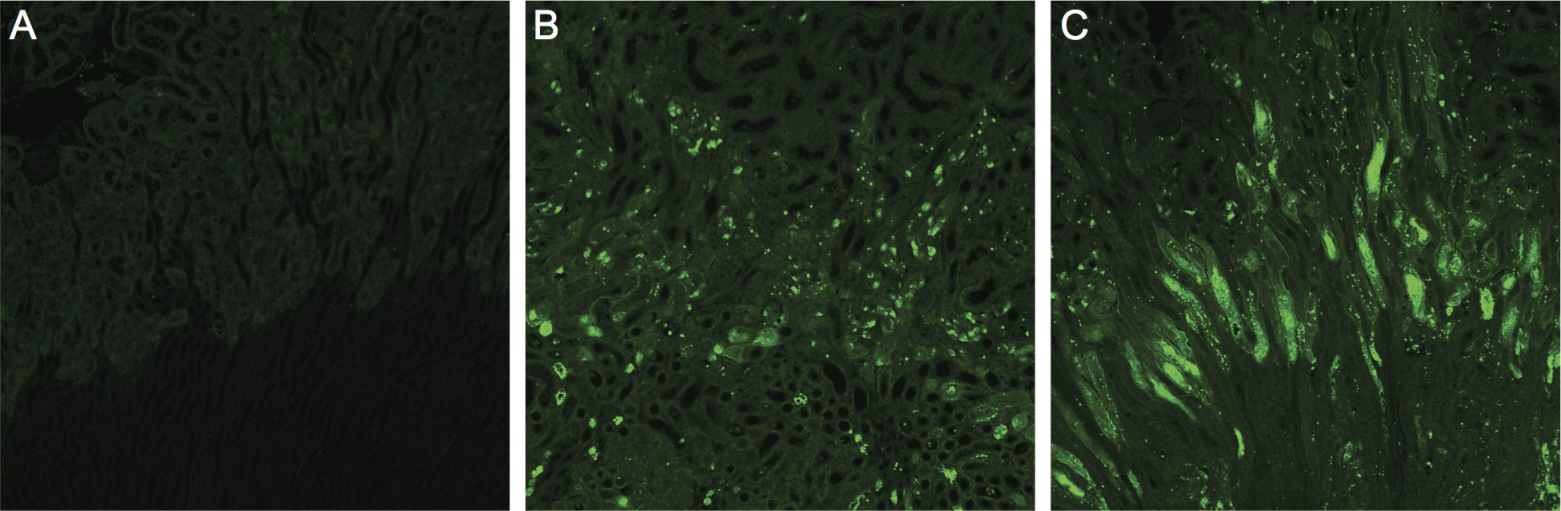
TUNEL stainings of mouse kidneys. A) Control kidney without IR damage. B) and C) Mouse kidneys 24 hours after IR. B) Control mouse on standard ad libitum chow and drinking water. C) Mouse treated with twice oral gavage of 250 μl of 10% GlcNAc solution.

To assess a potential effect of GlcNAc-treatment on apoptotic cell death after ischemia-reperfusion injury we performed TUNEL stainings. The assay showed extensive apoptosis after ischemia-reperfusion with no significant difference between groups (Fig 3).

### Effect of GlcNAc supplementation on kidney function after renal ischemia reperfusion injury

**Fig 4:**
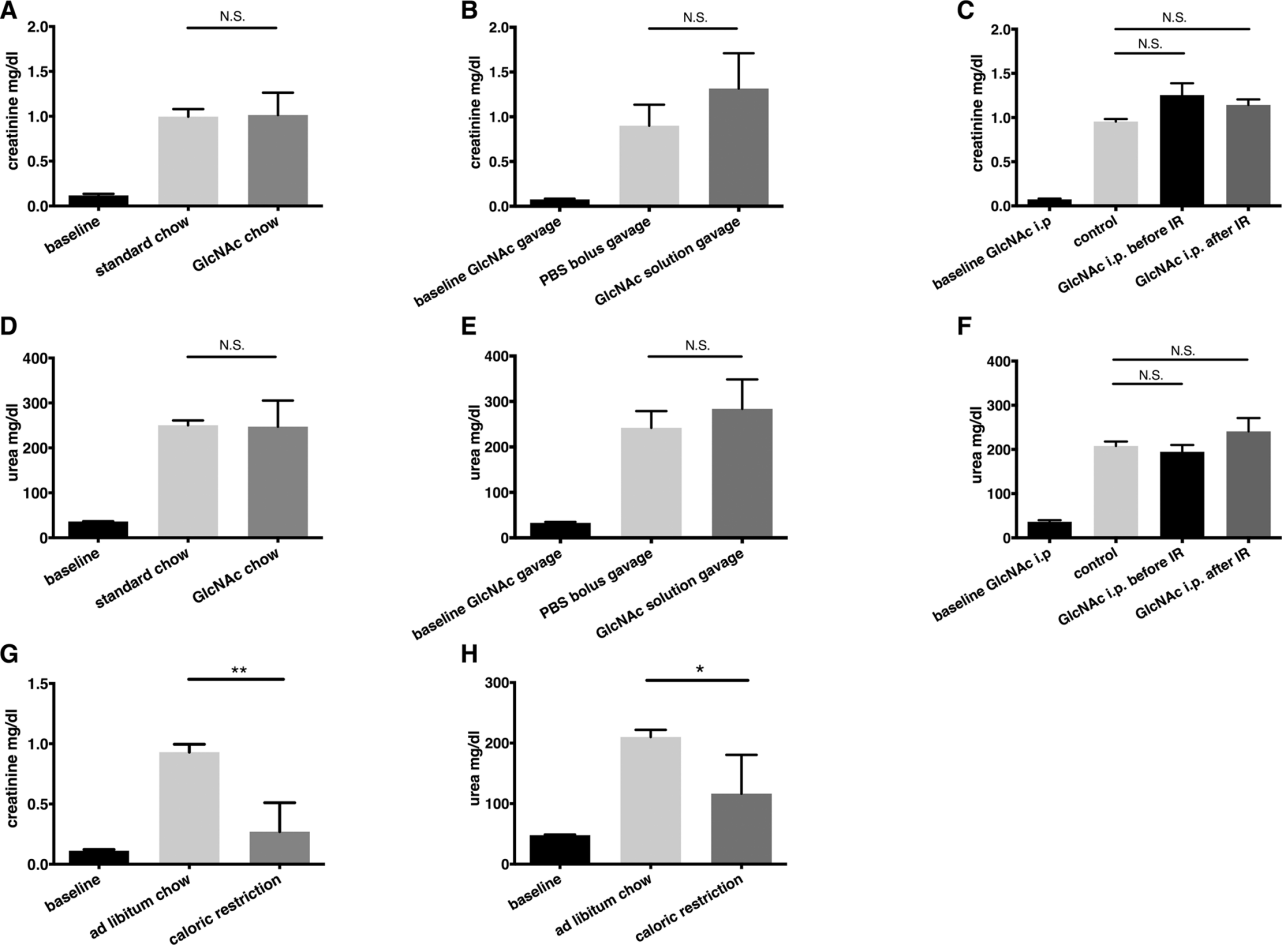
Kidney failure 24 hours after ischemia-reperfusion injury in preconditioned and control animals. Creatinine and urea values after unilateral nephrectomy followed by 40 minutes of ischemia and 24 hours of reperfusion (IR) of the contralateral kidney. Different dosing regimens for GlcNAc were used. A) and D) Mice were kept on a four-week ad libitum diet enriched with 0.5% GlcNAc (n = 17) or received standard chow ad libitum (n = 15). B) and E) Mice received a 250 μl PBS twice, 24 and 2 hours before IR by gavage (n = 5). In the verum group PBS contained 10% GlcNAc (n = 10). C) and F) Verum mice were injected 20 mg GlcNAc in PBS either one hour before surgery i.p (n = 3) or immediately at the end of ischemia at the beginning of reperfusion (n = 4) directly into the abdominal cavity. Baselines represent control animals from Fig 1A and 1B without ischemia-reperfusion injury A) and D) for untreated animals (n = 5), B) and E) for mice after gavage of 10% GlcNAc solution (n = 6) and C) and F) for mice after i.p. injection of 10 mg GlcNAc (n = 5). After four weeks of caloric restriction (n = 4) with standard chow mice are protected against ischemia-reperfusion damage compared to controls having ad libitum access to standard chow (n = 5) (G+H).

There are numerous publications showing that preconditioning by caloric restriction confers strong protection against renal ischemia-reperfusion injury [2,17]. In parallel with our GlcNAc experiments we included a group of mice that was objected to a 4 weeks interval of caloric restriction as a positive control for protection against ischemic kidney injury. Treatment consisted of a caloric restriction that equaled 60% of normal caloric intake. This simple approach led to a dramatic and robust attenuation of AKI in our model that consisted of a right nephrectomy followed by 40 minutes of ischemia of the left kidney and 24 hours of reperfusion (Fig 4G and 4H).

To test if GlcNAc protects against AKI, we subjected male BL6 mice to the same ischemia-reperfusion model as described above. There was no significant difference in creatinine or urea after ischemia-reperfusion injury in mice fed with chow containing 0.5% GlcNAc for 4 weeks compared to control animals fed with standard chow (Fig 4A and 4D).

Several different protocols for supplementing mice with GlcNAc have been used previously. While some groups administered GlcNAc via chow or drinking water for several weeks, as described above in our experiment, others have seen beneficial effects already after only a single bolus [18]. Since a four weeks approach did not show any benefit, we next decided to test if a short challenge with a high dose of GlcNAc would lead to renal protection. We fed mice a 10% GlcNAc solution via gavage 24 and 2 hours before renal ischemia-reperfusion injury. In contrast to published results that found protective effects of GlcNAc on cardiac and retinal ischemia-reperfusion injury we did not observe any protective effects after short-term treatment with GlcNAc in the kidney (Fig 4B and 4E). We also carried groups of sham animals along that only underwent uninephrectomy without contralateral ischemia-reperfusion injury. These animals showed a slight elevation in creatinine and urea values that was not different between animals after GlcNAc gavage and controls (S2 Fig).

To rule out problems with oral bioavailability as a reason for our negative results we repeated the former experiment with single GlcNAc i.p. injections. This approach did not lead to an improved outcome in the experimental animals (Fig 4C and 4F), either. Finally, Chen et al. just recently showed that administration of D-glucosamine (GlcN), another substrate of the HP, at the end of retinal ischemia provides even better protection than supplementation before damage [11]. In our model single i.p. administration of GlcNAc at the beginning of reperfusion did not lead to attenuation of kidney failure (Fig 4C and 4F).

Taken together, GlcNAc treatment by means of various protocols with respect to application method, timing, and treatment duration did not lead to nephroprotection in an ischemia-reperfusion injury mouse model. In contrast to GlcNAc administration, caloric restriction, an established preconditioning protocol, improved kidney function 24 hours after damage.

## Discussion

A growing body of evidence suggests improved cellular stress resistance mediated by activation of the HP or administration of the aminosugars produced by the HP. Chen and colleagues found that GlcN administration led to increased cell survival in a retinal ischemia rat model [11]. Jones et al. observed increased O-GlcNAcylation in myocardial tissue after ischemic preconditioning that was accompanied by cardioprotective effects [9,19].

In the present study we analyzed the effects of GlcNAc in the context of a murine renal ischemia-reperfusion injury model. In contrast to the above-mentioned studies regarding heart and retina, we did not find beneficial effects on the outcome after renal ischemia-reperfusion injury.

Current concepts suggest that O-GlcNAcylation is capable of mediating protective as well as harmful effects but the mechanisms defining the actual outcome are poorly understood. Altered O-GlcNAc cycling has been observed in a variety of chronic diseases like diabetes, cancer [20], cardiovascular disease, obesity [21] and Alzheimer’s disease [22]. There is evidence suggesting that acute increases in cardiomyocyte O-GlcNAcylation serve as a pro-survival signal. Zou and colleagues have shown that increased O-GlcNAcylation in hypovolemic cardiogenic shock leads to altered NF-kB signaling and improved cardiac function due to reduced inflammation [23]. Ngoh et al. provide evidence that O-GlcNAc transferase (OGT) overexpression in a murine ischemia-reperfusion injury model of the heart attenuated Ca^2+^ overload and ROS generation [24].

On the other hand, O-GlcNAcylation as typically seen in diabetes leads to altered mitochondrial protein O-GlcNAcylation, mislocalization of mitochondrial OGT and consecutively plays a role in mitochondrial dysfunction in the myocardium of diabetic rats [25]. Furthermore, despite its positive effects on cardiomyocytes, O-GlcNAcylation seems to have an unfavorable impact on vasomotor function, as increased O-GlcNAcylation has been shown to lead to vasoconstriction of the rat aorta [26].

Beyond O-GlcNAcylation, N-glycosylation and O-glycosylation that also act downstream of the HP might influence organ protective effects. N-glycosylation occurs at asparagine residues via step-by-step attachment of GlcNAc and other sugars in the endoplasmic reticulum. During maturation in the ER mainly secreted and cell surface proteins are N-glycosylated [27]. O-glycosylation is initiated in the Golgi and depends on a set of N-acetylgalactosaminyltransferases (GALNTs) [28]. It has been shown that CD95, also known as Fas or APO-1, is N-glycosylated at two extracellular sites [29]. CD95 signaling via procaspase-8 and various other downstream pathways regulates apoptosis and cell survival [30]. Shatnyeva and colleagues presented evidence for a role of N-glycosylation in the extent of CD95-induced apoptosis in cell culture. In their work N-deglycosylation of CD95 raised the threshold for apoptotic cell death [29]. Wagner et al., on the other hand, presented evidence for TRAIL/Apo2L mediated O-glycosylation dependent activation of caspase-8 [31]. These results underline the complexity of effects related to activation of the HP in the context of apoptotic cell death.

Additional parameters such as the duration of serum GlcNAc elevation, the peak levels of GlcNAc and the interplay of GlcNAcylation with further posttranslational modifications such as phosphorylation might also be of importance. Phosphorylation and O-GlcNAcylation can be found simultaneously but also function as competitors at the same or closely adjacent protein residues [32]. In view of these varying findings, one crucial factor determining benefit or harm might be timing and dosage. Duration of treatment, dosage and route of administration of aminosugars have been used under differing conditions in mice and rats depending on the target tissue and the pathophysiology of the underlying disease. Dosing ranged from 0.25 mg to 10 mg/g in experiments conducted in mice [7,33]. The published durations of treatment ranged from single bolus application in acute injury models (e.g. retinal ischemia) to repeated administrations that were used mostly in chronic disease (e.g. rheumatoid arthritis, experimental autoimmune encephalomyelitis) [34,35].

To address these issues we compared various protocols comprising oral and parenteral application as well as long and short term supplementation and also checked for adequate bioavailability. We found elevated circulating GlcNAc levels one hour after administration. Although GlcNAc has a sweet taste we could not observe differences in weight gain, when comparing GlcNAc supplemented to control animals, which argues against an increased caloric uptake. In addition, with respect to an envisioned possible use in the clinical setting, we reckon that the here-investigated strategies would seem most appropriate at least with regard to timing.

It should be taken into account that interspecies differences or tissue specific effects might explain the negative results of our study, as positive effects of amino sugars in the context of IR injury were often observed in models using rats or cell culture [24,36]. Thus, it cannot be excluded that renal protection after IR might occur in rat or mouse strains other than C57BL/6J.

In summary, our study is the first to test GlcNAc for preconditioning in a murine model of renal ischemia-reperfusion injury. Unfortunately, with none of the employed protocols we were able to demonstrate a beneficial impact on renal protection. However, protection was successfully achieved using caloric restriction, which excludes protocol-specific problems that would render prevention of AKI impossible. We therefore have to conclude that GlcNAc supplementation does not seem to be a promising approach for clinical utilization at this time.

## Supporting Information

S1 Fig Experimental protocols. A long term (A) and a short term (B) oral GlcNAc supplementation as well as a single shot intraperitoneal (i.p.) administration (C) were employed. I.p. application took place either one hour before ischemia (C upper panel) or directly at the end of it (C lower panel). All animals underwent right nephrectomy followed by 40 minutes of left renal ischemia (I) and were sacrificed 24 hours after restoration of perfusion (✝).
(TIFF)

S2 Fig Effect of sham surgery on creatinine and urea values. Animals were preconditioned by twice GlcNAc or PBS (controls) gavage and underwent unilateral nephrectomy, followed by mobilization of the contralateral renal pedicle without IR afterwards. Serum creatinine and urea values were assessed at baseline and 24 hours after reperfusion.
(TIFF)

## References

[1] Rewa O, Bagshaw SM. Acute kidney injury—epidemiology, outcomes and economics. Nature Publishing Group. Nature Publishing Group; 2014;10: 193–207. 10.1038/nrneph.2013.282 24445744

[2] Mitchell JR, Verweij M, Brand K, van de Ven M, Goemaere N, van den Engel S, et al Short-term dietary restriction and fasting precondition against ischemia reperfusion injury in mice. Aging Cell. 2010;9: 40–53. 10.1111/j.1474-9726.2009.00532.x 19878145 PMC3412229

[3] Bernhardt WM, Câmpean V, Kany S, Jürgensen J-S, Weidemann A, Warnecke C, et al Preconditional activation of hypoxia-inducible factors ameliorates ischemic acute renal failure. Journal of the American Society of Nephrology. American Society of Nephrology; 2006;17: 1970–1978. 10.1681/ASN.2005121302 16762988

[4] Mehta R, Steinkraus KA, Sutphin GL, Ramos FJ, Shamieh LS, Huh A, et al Proteasomal regulation of the hypoxic response modulates aging in C. elegans. Science. 2009;324: 1196–1198. 10.1126/science.1173507 19372390 PMC2737476

[5] Müller R-U, Fabretti F, Zank S, Burst V, Benzing T, Schermer B. The von Hippel Lindau Tumor Suppressor Limits Longevity. Journal of the American Society of Nephrology. 2009;20: 2513–2517. 10.1681/ASN.2009050497 19797165 PMC2794223

[6] Zachara NE. The roles of O-linked -N-acetylglucosamine in cardiovascular physiology and disease. Am J Physiol Heart Circ Physiol. 2012;302: H1905–H1918. 10.1152/ajpheart.00445.2011 22287582 PMC3362101

[7] Weimer S, Priebs J, Kuhlow D, Groth M, Priebe S, Mansfeld J, et al D-Glucosamine supplementation extends life span of nematodes and of ageing mice. Nature Communications. Nature Publishing Group; 2014;5: 1–12. 10.1038/ncomms4563 PMC3988823 24714520

[8] Denzel MS, Storm NJ, Gutschmidt A, Baddi R, Hinze Y, Jarosch E, et al Hexosamine Pathway Metabolites Enhance Protein Quality Control and Prolong Life. Cell. Elsevier Inc; 2014;156: 1167–1178. 10.1016/j.cell.2014.01.061 24630720

[9] Wang ZV, Deng Y, Gao N, Pedrozo Z, Li DL, Morales CR, et al Spliced X-Box Binding Protein 1 Couples the Unfolded Protein Response to Hexosamine Biosynthetic Pathway. Cell. Elsevier; 2014;156: 1179–1192. 10.1016/j.cell.2014.01.014 24630721 PMC3959665

[10] Jensen RV, Johnsen J, Kristiansen SB, Zachara NE, Bøtker HE. Ischemic preconditioning increases myocardial O-GlcNAc glycosylation. Scand Cardiovasc J. 2013;47: 168–174. 10.3109/14017431.2012.756984 23301939

[11] Chen Y-J, Huang Y-S, Chen J-T, Chen Y-H, Tai M-C, Chen C-L, et al Protective effects of glucosamine on oxidative-stress and ischemia/reperfusion-induced retinal injury. Invest Ophthalmol Vis Sci. 2015;56: 1506–1516. 10.1167/iovs.14-15726 25655796

[12] Khan NA, Susa D, van den Berg JW, Huisman M, Ameling MH, van den Engel S, et al Amelioration of renal ischaemia-reperfusion injury by synthetic oligopeptides related to human chorionic gonadotropin. Nephrology Dialysis Transplantation. 2009;24: 2701–2708. 10.1093/ndt/gfp369 19633318

[13] Tirapelli LF, Barione DF, Trazzi BFM, Tirapelli DPC, Novas PC, Silva CS, et al Comparison of two models for evaluation histopathology of experimental renal ischemia. Transplantation Proceedings. 2009;41: 4083–4087. 10.1016/j.transproceed.2009.09.061 20005345

[14] Goujon JM, Hauet T, Menet E, Levillain P, Babin P, Carretier M. Histological evaluation of proximal tubule cell injury in isolated perfused pig kidneys exposed to cold ischemia. Journal of Surgical Research. 1999;82: 228–233. 10.1006/jsre.1998.5526 10090834

[15] Takahashi M, Inoue K, Yoshida M, Morikawa T, Shibutani M, Nishikawa A. Lack of chronic toxicity or carcinogenicity of dietary N-acetylglucosamine in F344 rats. Food and Chemical Toxicology. Elsevier Ltd; 2009;47: 462–471. 10.1016/j.fct.2008.12.002 19103248

[16] Robertson LT, Mitchell JR. Benefits of short-term dietary restriction in mammals. Experimental Gerontology. 2013;48: 1043–1048. 10.1016/j.exger.2013.01.009 23376627 PMC3745522

[17] Lempiäinen J, Finckenberg P, Mervaala EE, Sankari S, Levijoki J, Mervaala EM. Caloric restriction ameliorates kidney ischaemia/reperfusion injury through PGC-1α-eNOS pathway and enhanced autophagy. Acta Physiol. 2013;208: 410–421. 10.1111/apha.12120 23710679

[18] Hwang S-Y, Shin J-H, Hwang J-S, Kim S-Y, Shin J-A, Oh E-S, et al Glucosamine exerts a neuroprotective effect via suppression of inflammation in rat brain ischemia/reperfusion injury. Glia. 2010;58: 1881–1892. 10.1002/glia.21058 20737476

[19] Jones SP, Zachara NE, Ngoh GA, Hill BG, Teshima Y, Bhatnagar A, et al Cardioprotection by N-Acetylglucosamine Linkage to Cellular Proteins. Circulation. 2008;117: 1172–1182. 10.1161/CIRCULATIONAHA.107.730515 18285568

[20] Ferrer CM, Lynch TP, Sodi VL, Falcone JN, Schwab LP, Peacock DL, et al O-GlcNAcylation regulates cancer metabolism and survival stress signaling via regulation of the HIF-1 pathway. Molecular Cell. 2014;54: 820–831. 10.1016/j.molcel.2014.04.026 24857547 PMC4104413

[21] Medford HM, Chatham JC, Marsh SA. Chronic ingestion of a Western diet increases O-linked-β-N-acetylglucosamine (O-GlcNAc) protein modification in the rat heart. Life Sciences. Elsevier Inc; 2012;90: 883–888. 10.1016/j.lfs.2012.04.030 22575823 PMC3372663

[22] Zhu Y, Shan X, Yuzwa SA, Vocadlo DJ. The emerging link between O-GlcNAc and Alzheimer disease. J Biol Chem. American Society for Biochemistry and Molecular Biology; 2014;289: 34472–34481. 10.1074/jbc.R114.601351 25336656 PMC4263855

[23] Zou L, Yang S, Champattanachai V, Hu S, Chaudry IH, Marchase RB, et al Glucosamine improves cardiac function following trauma-hemorrhage by increased protein O-GlcNAcylation and attenuation of NF-{kappa}B signaling. Am J Physiol Heart Circ Physiol. 2009;296: H515–23. 10.1152/ajpheart.01025.2008 19098112 PMC2643896

[24] Ngoh GA, Watson LJ, Facundo HT, Jones SP. Augmented O-GlcNAc signaling attenuates oxidative stress and calcium overload in cardiomyocytes. Amino Acids. Springer Vienna; 2011;40: 895–911. 10.1007/s00726-010-0728-7 20798965 PMC3118675

[25] Banerjee PS, Ma J, Hart GW. Diabetes-associated dysregulation of O-GlcNAcylation in rat cardiac mitochondria. Proc Natl Acad Sci U S A. 2015;112: 6050–6055. 10.1073/pnas.1424017112 25918408 PMC4434690

[26] Lima VV, Giachini FRC, Carneiro FS, Carneiro ZN, Fortes ZB, Carvalho MHC, et al Increased vascular O-GlcNAcylation augments reactivity to constrictor stimuli—Vasoactive Peptide Symposium. J Am Soc Hypertens. 2008;2: 410–417. 10.1016/j.jash.2008.06.001 19884969 PMC2630260

[27] Parodi AJ. Role of N-oligosaccharide endoplasmic reticulum processing reactions in glycoprotein folding and degradation. Biochem J. 2000;348 Pt 1: 1–13. 10.1042/bj3480001 10794707 PMC1221029

[28] Schjoldager KTBG, Clausen H. Site-specific protein O-glycosylation modulates proprotein processing—deciphering specific functions of the large polypeptide GalNAc-transferase gene family. Biochim Biophys Acta. 2012;1820: 2079–2094. 10.1016/j.bbagen.2012.09.014 23022508

[29] Shatnyeva OM, Kubarenko AV, Weber CEM, Pappa A, Schwartz-Albiez R, Weber ANR, et al Modulation of the CD95-Induced Apoptosis: The Role of CD95 N-Glycosylation. Pastore A, editor. PLoS ONE. Public Library of Science; 2011;6: e19927 10.1371/journal.pone.0019927 21625644 PMC3097226

[30] Fouqué A, Debure L, Legembre P. The CD95/CD95L signaling pathway: a role in carcinogenesis. Biochim Biophys Acta. 2014;1846: 130–141. 10.1016/j.bbcan.2014.04.007 24780723

[31] Wagner KW, Punnoose EA, Januario T, Lawrence DA, Pitti RM, Lancaster K, et al Death-receptor O-glycosylation controls tumor-cell sensitivity to the proapoptotic ligand Apo2L/TRAIL. Nature Medicine. Nature Publishing Group; 2007;13: 1070–1077. 10.1038/nm1627 17767167

[32] Wang Z, Gucek M, Hart GW. Cross-talk between GlcNAcylation and phosphorylation: site-specific phosphorylation dynamics in response to globally elevated O-GlcNAc. Proc Natl Acad Sci U S A. 2008;105: 13793–13798. 10.1073/pnas.0806216105 18779572 PMC2544533

[33] Zhang G-X, Yu S, Gran B, Rostami A. Glucosamine abrogates the acute phase of experimental autoimmune encephalomyelitis by induction of Th2 response. The Journal of Immunology. 2005;175: 7202–7208. 16301624 10.4049/jimmunol.175.11.7202

[34] Azuma K, Osaki T, Wakuda T, Tsuka T, Imagawa T, Okamoto Y, et al Suppressive Effects of N-Acetyl-d-Glucosamine on Rheumatoid Arthritis Mouse Models. Inflammation. 2012;35: 1462–1465. 10.1007/s10753-012-9459-0 22434264

[35] Grigorian A, Araujo L, Naidu NN, Place DJ, Choudhury B, Demetriou M. N-Acetylglucosamine Inhibits T-helper 1 (Th1)/T-helper 17 (Th17) Cell Responses and Treats Experimental Autoimmune Encephalomyelitis. Journal of Biological Chemistry. 2011;286: 40133–40141. 10.1074/jbc.M111.277814 21965673 PMC3220534

[36] Liu J, Marchase RB, Chatham JC. Glutamine-induced protection of isolated rat heart from ischemia/reperfusion injury is mediated via the hexosamine biosynthesis pathway and increased protein O-GlcNAc levels. Journal of Molecular and Cellular Cardiology. 2007;42: 177–185. 10.1016/j.yjmcc.2006.09.015 17069847 PMC1779903

