# Supplementary figures and images for: Oral Supplementation of Glucosamine Fails to Alleviate Acute Kidney Injury in Renal Ischemia-Reperfusion Damage
Source: PLoS One. 2016 Aug 24;11(8):e0161315. doi: 10.1371/journal.pone.0161315 (PMC4996512; doi:10.1371/journal.pone.0161315)

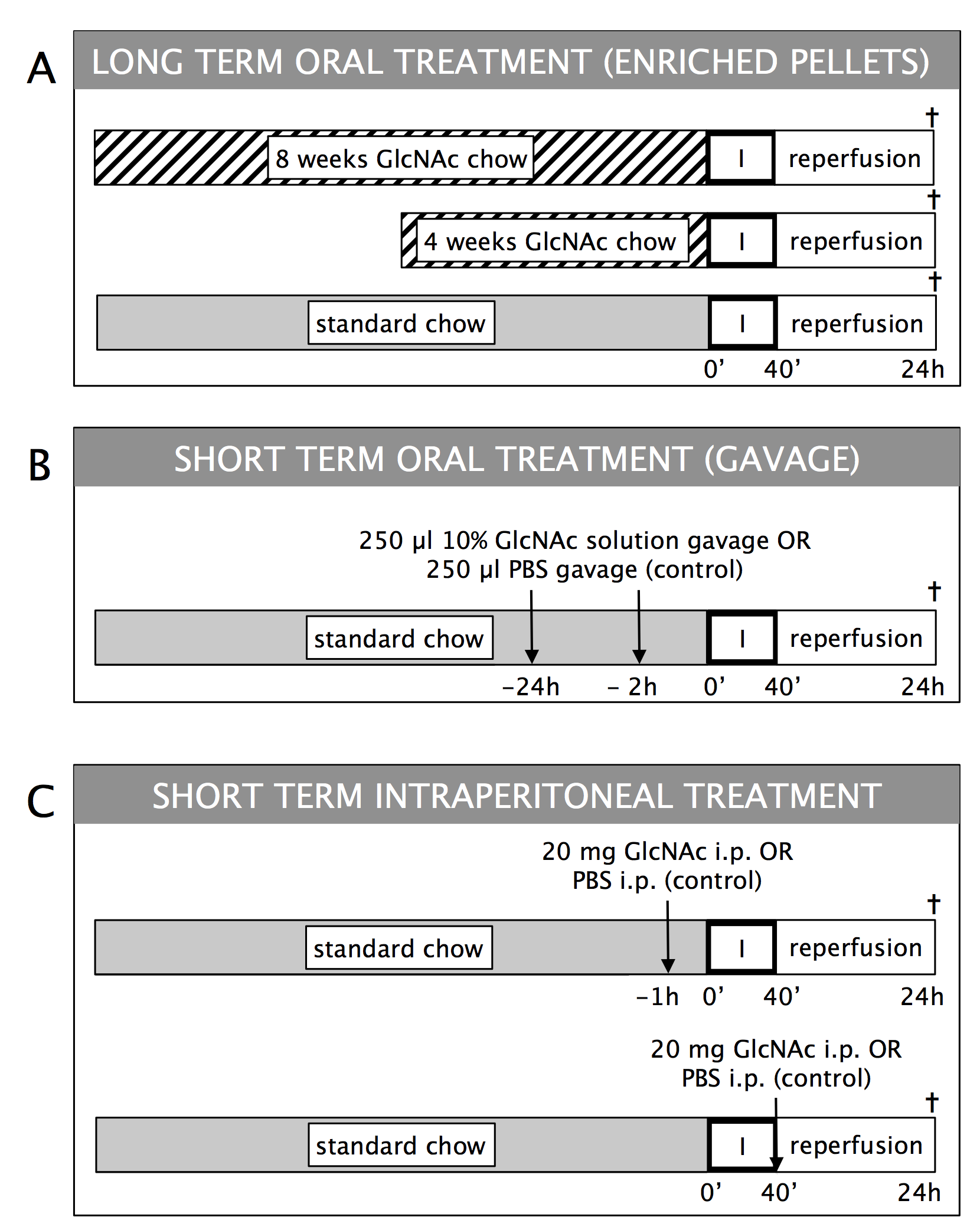

Supplement: S1 Fig — A long term (A) and a short term (B) oral GlcNAc supplementation as well as a single shot intraperitoneal (i.p.) administration (C) were employed. I.p. application took place either one hour before ischemia (C upper panel) or directly at the end of it (C lower panel). All animals underwent right nephrectomy followed by 40 minutes of left renal ischemia (I) and were sacrificed 24 hours after restoration of perfusion (✝). (TIFF) [file pone.0161315.s001.tiff]

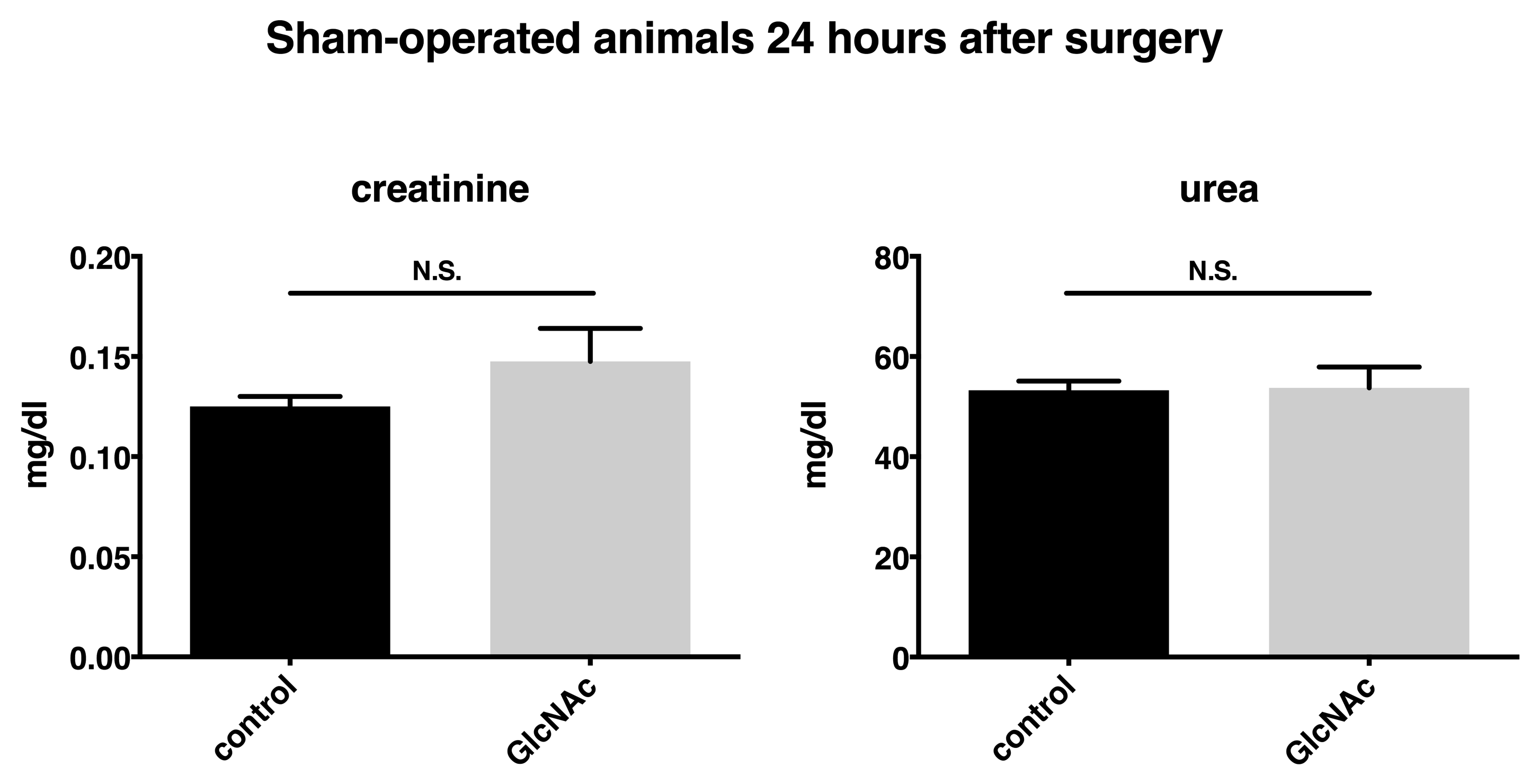

Supplement: S2 Fig — Animals were preconditioned by twice GlcNAc or PBS (controls) gavage and underwent unilateral nephrectomy, followed by mobilization of the contralateral renal pedicle without IR afterwards. Serum creatinine and urea values were assessed at baseline and 24 hours after reperfusion. (TIFF) [file pone.0161315.s002.tiff]
